# Comparison of efficacy and safety of mirabegron and vibegron in the treatment of Overactive Bladder (OAB) in older women: A systematic review and meta-analysis

**DOI:** 10.1371/journal.pone.0317550

**Published:** 2025-04-08

**Authors:** Jiankun Zhang, Junpeng Chi, Keyuan Lou, Junjie Zhao, Feng Gao, Yuanshan Cui

**Affiliations:** 1 School of Clinical Medicine, Shandong Second Medical University, Weifang, China; 2 Department of Urology, Yantai Yuhuangding Hospital, Yantai, China; 3 Department of Urology, Weifang People’s Hospital, WeiFang, China; Weill Cornell Medical College, UNITED STATES OF AMERICA

## Abstract

**Background:**

After the introduction of anticholinergic drugs for the treatment of overactive bladder (OAB), the discovery of β-adrenergic agonists has helped reduce the side effects associated with the former. Currently, the two available medications, mirabegron and vibegron, are β-adrenergic agonists. However, clinical practitioners are still faced with the dilemma of which drug to choose.

**Objective:**

To analyze and compare the efficacy and adverse effects of the two medications.

**Methods:**

A literature search was conducted to identify randomized controlled trials using mirabegron and vibegron for the treatment of OAB. Databases such as PubMed, Web of Science, Cochrane Library, and Embase were searched. The search cutoff date was July 25 2024. Data extraction and quality assessment were performed using standardized methods. A meta-analysis was then conducted using RevMan software and a random-effects model, with studies weighted according to sample size and variance. Heterogeneity was assessed using the I² statistic. All statistical analyses were performed using RevMan, and results were presented as effect sizes (e.g., mean difference or risk ratio).

**Results:**

Three randomized controlled trials compared the safety and efficacy of mirabegron and vibegron head-to-head, involving 368 patients. The trials, each lasting 8 or 12 weeks. The trials compared the changes in various indices of the OABSS (Overactive Bladder Symptom Score) between the two drugs. The statistical methods used in the analysis included Mean Difference (MD), 95% Confidence Interval (CI), p-value, and I² statistic. For OABSS: MD =  0.38, 95% CI =  − 0.19 to 0.95, p =  0.28, I² =  21%; for Q1: MD =  0.08, 95% CI =  − 0.01 to 0.26, p =  0.31, I² =  4%; for Q2: MD =  0.08, 95% CI =  − 0.21 to 0.37, p =  0.67, I² =  0%; for Q3: MD =  0.05, 95% CI =  − 0.45 to 0.56, p =  0.90, I² =  0%; for Q4: MD =  − 0.21, 95% CI =  − 0.68 to 0.27, p =  0.35, I² =  0%. The relative risk (RR) of adverse effects between the two drugs was: RR =  0.87, 95% CI =  0.57 to 1.34, p =  0.27, I² =  25%; for constipation: RR =  0.73, 95% CI =  0.37 to 1.43, p =  0.27, I² =  25%; and for dry mouth: RR =  0.98, 95% CI =  0.42 to 2.30, p =  0.78, I² =  0%.

**Conclusion:**

There appears to be no statistically significant difference in efficacy and safety between mirabegron and vibegron for OAB patients. Further high-quality prospective studies are needed to confirm these results.

## Introducation

Overactive bladder (OAB) is a condition characterized by symptoms such as urgency, incontinence, and nocturia, which can arise from aging and neurological disorders [1,2]. The prevalence of OAB is 16.8% to 43% in Western countries and 20.8% in Asian countries, with a higher incidence in women than in men [3]. Due to its chronic nature, difficulty in symptom relief, and significant physical and psychological impact, OAB can lead to a reduction in the quality of life for affected individuals.

To date, anticholinergic medications (such as oxybutynin and trospium) and β-adrenergic agonists (such as mirabegron) have been widely used to treat OAB, including both non-neurogenic overactive bladder (non-NOAB) and neurogenic overactive bladder (NOAB). [4] These medications typically provide significant symptom relief, though their efficacy and adverse effects can vary between individuals. Mirabegron, in particular, has efficacy similar to that of anticholinergic drugs, but with fewer adverse effects, such as dry mouth [5,6].

With the discovery of the new drug vibegron, there is increasing interest in determining which medication offers better efficacy for treating OAB. Both mirabegron [7] and vibegron [8] are new β3-adrenergic receptor agonists (β3-AR). While there have been studies that indirectly compare mirabegron and vibegron, no direct comparison of their efficacy and safety has been conducted. In this study, we performed a head-to-head direct comparison of mirabegron and vibegron for the treatment of OAB in older women, evaluating their efficacy and safety.

## Methods

This meta-analysis is reported in accordance with the Preferred Reporting Items for Systematic Reviews and Meta-Analyses (PRISMA) statement and was registered at International Prospective Register of Systematic Reviews (number: CRD42024583601).

### Search strategy

We conducted searches using PubMed, Web of Science, Embase and Cochrane Library. Articles were retrieved up to July 25, 2024, with no language restrictions. The search terms used were “mirabegron” and “vibegron,” including merged text and MeSH terms. Results included 11 articles from the Cochrane Library, 38 from PubMed, 15 from Web of Science, and 24 from Embase. The complete search strategy for PubMed was: (((mirabegron) OR (((((2-(2-aminothiazol-4-yl)-4’-(2-((2-hydroxy-2-phenylethyl)amino)ethyl)acetanilide) OR (YM-178)) OR (Betmiga)) OR (YM 178)) OR (Betanis))) OR (mirabegron[Title/Abstract])) AND (((vibegron) OR (MK-4618)) OR (N-(4-((5-(hydroxy(phenyl)methyl)pyrrolidin-2-yl)methyl)phenyl)-4-oxo-4,6,7,8-tetrahydropyrrolo(1,2-a)pyrimidine-6-carboxamide)).

### Inclusion and exclusion criteria

We applied strict inclusion and exclusion criteria. We selected randomized clinical trials comparing the two treatment strategies, mirabegron and vibegron, in older female OAB patients with an intervention period of at least 6 weeks and reporting Overactive Bladder Symptom Score (OABSS), a validated tool used to assess the severity of symptoms associated with OAB, including frequency, urgency, nocturia, and incontinence. This study primarily included women aged 50 years and older, or those who were postmenopausal. We defined women aged 50 years and above as “older women” which is consistent with the commonly used definitions in many epidemiological studies and clinical trials. All included studies selected participants within this age range to ensure the homogeneity and representativeness of the study results. Exclusion criteria included: observational and retrospective studies; studies with an intervention period of less than 6 weeks; and studies not assessing the effects of mirabegron and vibegron. Non-comparative studies, those without outcome reports, missing data, as well as reviews, meta-analyses, and conference abstracts were excluded.

**Fig 1:**
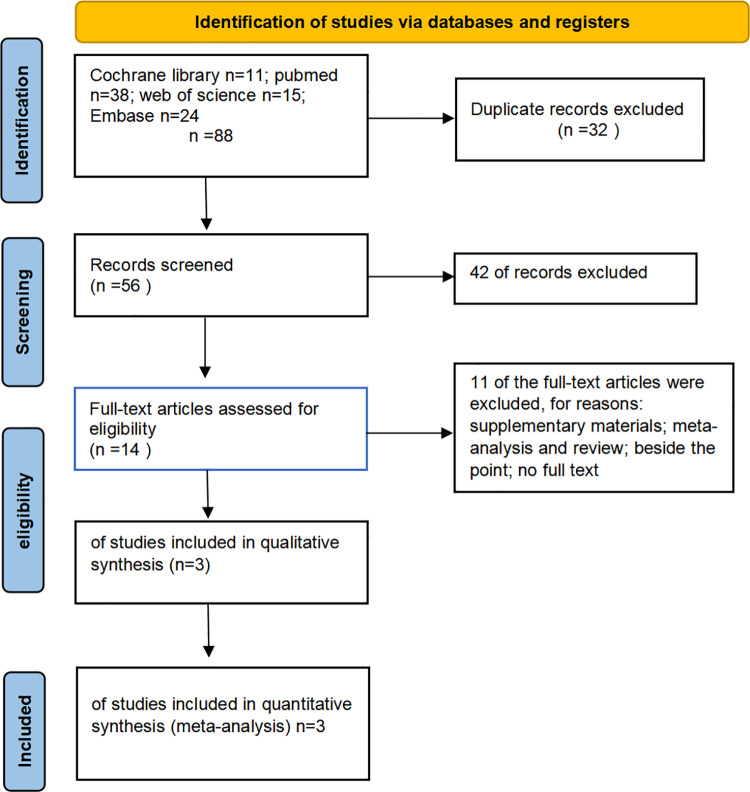
PRISMA Flow Diagrame.

Two independent researchers reviewed the study titles and abstracts, retrieving full-text studies that met the inclusion criteria for detailed evaluation. The trials selected for in-depth analysis were reviewed by two researchers, with disagreements resolved by a third researcher. (Fig 1)

### Data extraction

We extracted the following data from each selected study, where the inclusion criteria specified participants aged over 50 years: (1) First author and publication year; (2) Trial design and sample size; (3) Study protocol; (4) Country where the study was conducted; (5) Changes in OABSS scores (including changes in Q1-Q4 scores), post-void residual (PVR), and adverse effects. Data were independently extracted into tables by two reviewers, with any discrepancies resolved through discussion. (Table 1)

**Table 1:** The characteristics of the included studies.

Number	First author	Duration of the study	Year	country of publication	Study Design	Population characteristics	Sample Size	Arms	Intervention
1	Wada N, Mizunaga M	2019.1- 2022.12	2024	Japan	Randomized Controlled Trial	Female patients;OAB patient	67	Mirabegron(N = 33) Vibegron(N = 34)	M OR V (8weeks)
2	Sato H, Otsuka S	2019.12- 2022.2	2023	Japan	Randomized Controlled Trial	Female patients;OAB patient	102	Mirabegron(N = 50) Vibegron(N = 52)	M OR V (8weeks)
3	Kinjo M, Masuda K	2019.1- 2021.12	2023	Japan	Randomized Controlled Trial	Female patients;OAB patient	199	Mirabegron(N = 97) Vibegron(N = 102)	M OR V (12weeks)

### Quality assessment

**Fig 2:**
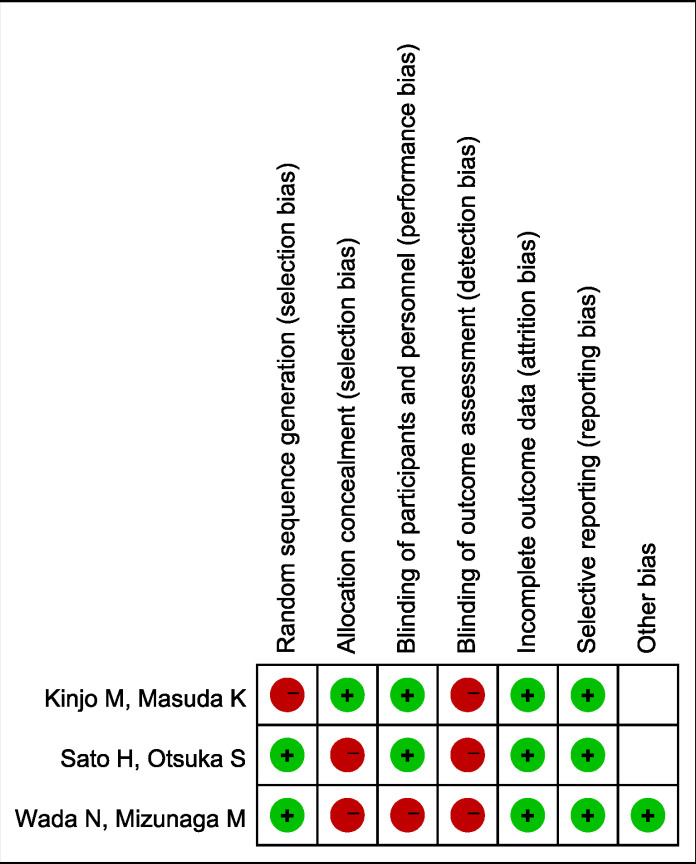
Quality assessment of individual study.

The risk of bias for each study was assessed using the Cochrane risk-of-bias tool for randomized controlled trials (RCTs). Biases were categorized into selection bias, performance bias, detection bias, attrition bias, reporting bias, and other biases, and were classified as low risk, high risk, or unclear risk. Two independent reviewers assessed the risk of bias according to the PRISMA guidelines. (Fig 2)

### Statistical analysis

Data synthesis and statistical analysis were performed using RevMan 5.4.1. For continuous outcomes (difference between pre- and post-treatment OABSS), mean differences (MD) and 95% confidence intervals (CI) were calculated. For dichotomous outcomes (complications), risk ratios (RR) and 95% CI were computed. This study examined the incidence of adverse events at 16 weeks and 12 weeks post-treatment. In cases where certain studies did not report the standard deviation (SD) or standard error (SE), missing data were estimated using other available statistical information (such as mean and standard error), or, if necessary, obtained directly from the study authors.

By analyzing the individual dimensions of the OABSS, we were able to assess the differential effects of mirabegron and vibegron on various OAB symptoms. This approach allowed us to gain a deeper understanding of how each drug targets specific symptoms. Separating the analysis of Q1-Q4 enhanced the sensitivity of our study, enabling us to detect subtle differences in drug efficacy that may not have been apparent in a combined analysis.

PVR was used as a secondary outcome to evaluate the effect of treatment on bladder function. To assess heterogeneity between studies, the I² statistic was used. The I² value represents the proportion of variability in effect sizes due to actual differences rather than random error. When the I² value was below 50%, a fixed-effect model was used, assuming the effect sizes were consistent; when the I² value exceeded 50%, a random-effects model was used to account for differences between studies.

Funnel plots were used to assess publication bias. A symmetrical funnel plot suggests no significant bias, while asymmetry, particularly if small studies cluster on one side, may indicate the presence of publication bias. Statistical significance was assessed using p-values (<0.05). A p-value less than 0.05 was considered statistically significant, indicating that the observed result was unlikely to be due to random error.

### Explanation of MD and I² statistic

MD is used to measure the average difference between two groups on a continuous outcome. In this study, MD was used to compare the improvement in OABSS scores between the treatment and control groups. For example, if the treatment group improves by 5 points and the control group improves by 3 points, the MD would be 2 points, indicating a greater improvement with the treatment group. The MD helps assess the magnitude and practical significance of the treatment effect.

The I² statistic is used to assess heterogeneity among studies in a meta-analysis. It represents the proportion of variability in effect sizes that is due to real differences between studies, rather than random error. When the I² value is close to 0%, it indicates that differences between studies are primarily due to random error. A higher I² value (typically above 50%) suggests significant heterogeneity, which may be attributed to differences in study design, patient populations, or interventions. The level of I² helps determine which model should be used for analysis. If I² is low, a fixed-effect model is typically employed; if I² is high, a random-effects model is used.

## Results

### Characteristics of the individual studies

A search of the database identified 88 articles potentially eligible for inclusion in our meta-analysis. Based on the inclusion and exclusion criteria, 85 articles were excluded after reviewing their titles and abstracts. A total of 3 studies met the inclusion criteria and were included in the meta-analysis [9–11]. The selection process and reasons for exclusion are detailed in a flowchart. In all three included RCTs, the drug dosage was 50 mg/day, with both mirabegron and vibegron administered at this dose. All included studies provided relevant information on changes in OABSS, reported adverse events during mirabegron or vibegron treatment, and 2 studies described changes in residual urine volume post-treatment.

### Quality of individual studies

All 3 studies were RCTs and described their randomization processes. Each study included power calculations to determine the optimal sample size. According to the revised RCT quality assessment tool from the Cochrane Library, most of the included studies were classified as having “low risk of bias.” (Fig 2) The funnel plot (Supplement 1A) provided a qualitative estimation of the publication bias across the included studies. The plot appeared symmetric, indicating no evidence of publication bias. Similarly, the funnel plot for the risk ratio (RR) of adverse events (Supplement 1B) also showed symmetry, suggesting that publication bias was not a significant issue in the included studies.

### OABSS

**Fig 3:**
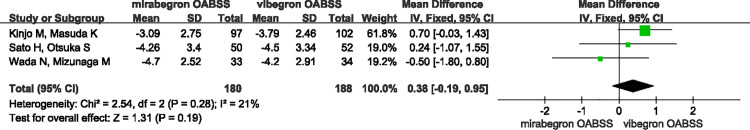
Forest plot of the mean changes in OABSS before and after treatment.

The three RCTs included a total of 368 participants (180 in the mirabegron group and 188 in the vibegron group) and provided data on the mean changes in OABSS before and after treatment (Fig 3). In the OABSS analysis, mirabegron showed less reduction compared to vibegron (MD =  0.38, 95% confidence interval [CI] =  − 0.19 to 0.95, p =  0.28, I² =  21%). This result indicates that there is no statistically significant difference between mirabegron and vibegron in reducing OABSS.

### Key symptoms assessed in the OABSS

**Fig 4:**
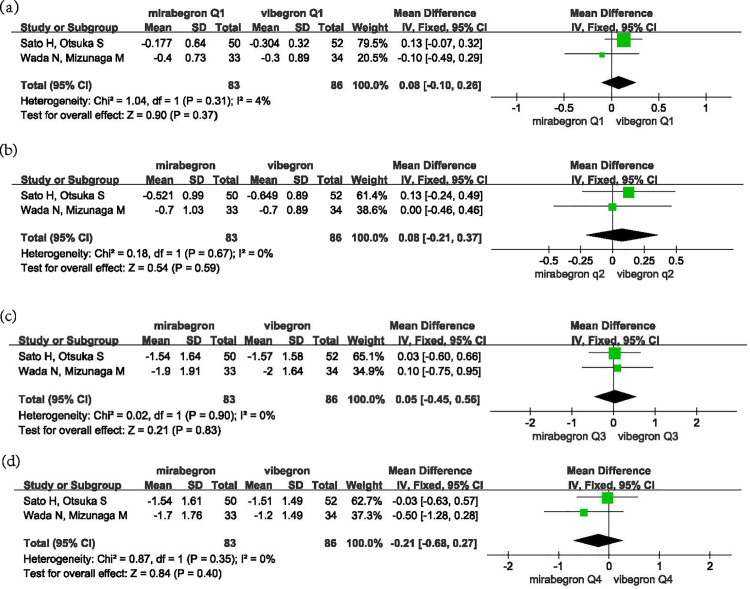
Forest plot of the mean changes in Q1-Q4 before and after treatment.

We further analyzed the four components of the OABSS score, specifically Q1-Q4, which include the average frequency of daytime symptoms (Q1), nocturia (Q2), urgency (Q3), and urgency urinary incontinence (Q4) post-treatment. The results showed: For Q1, the mean difference (MD) was 0.08, with a 95% confidence interval (CI) of − 0.01 to 0.26, p =  0.31, and I² =  4%; For Q2, the MD was 0.08, with a 95% CI of − 0.21 to 0.37, p =  0.67, and I² =  0%; For Q3, the MD was 0.05, with a 95% CI of − 0.45 to 0.56, p =  0.90, and I² =  0%; For Q4, the MD was − 0.21, with a 95% CI of − 0.68 to 0.27, p =  0.35, and I² =  0% (Fig 4). For Q1-Q4, the changes in scores before and after medication for both drugs did not show statistically significant differences.

### Post-void residual

**Fig 5:**
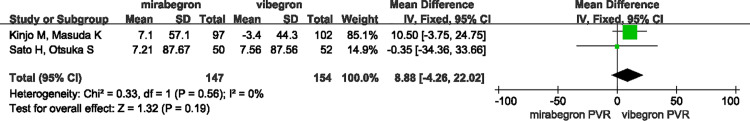
Forest plot of the mean changes in PVR before and after treatment.

Data on post-void residual (PVR) volume were obtained from two randomized controlled trials (RCTs), involving a total of 301 participants (147 in the mirabegron group and 154 in the vibegron group). The overall pooled analysis showed no statistically significant difference in PVR between the two treatment groups (mean difference [MD] =  8.88, 95% confidence interval [CI] =  − 4.26 to 22.02, P =  0.56, I² =  0%) (Fig 5).

In the first study, the mirabegron group had a baseline PVR of 29.0 ±  37.9 mL, which increased to 36.1 ±  42.6 mL after 12 weeks (P =  0.516). The vibegron group had a baseline PVR of 26.5 ±  34.4 mL, which decreased to 23.1 ±  27.8 mL after 12 weeks (P =  0.252). ^10^In the second study, the change in PVR was reported as a mean difference of 7.21 mL (95% CI: − 17.1 to 31.5, n =  50) [11].

### Safety

**Fig 6:**
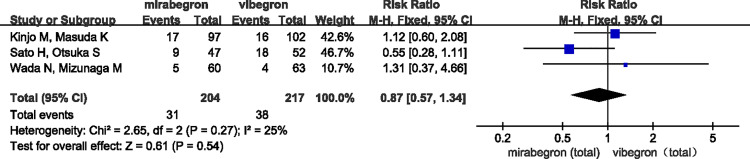
Forest plot of RR of adverse events after medication.

**Fig 7:**
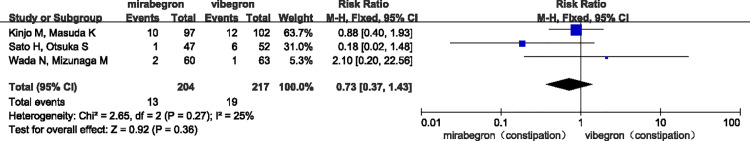
Forest plot of RR of the incidence of constipation.

**Fig 8:**
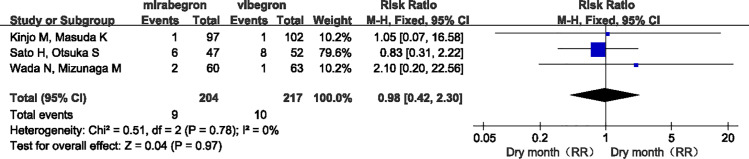
Forest plot of RR of the incidence of dry mouth.

The overall difference was not statistically significant (RR =  0.87, 95% confidence interval [CI] =  0.57 to 1.34, P =  0.27, I² =  25%) (Fig 6). Specifically, the incidence of constipation was lower with mirabegron compared to vibegron, but the difference was not statistically significant (RR =  0.73, 95% CI =  0.37 to 1.43, P =  0.27, I² =  25%) (Fig 7). There appeared to be no difference between the two drugs in the incidence of dry mouth (RR =  0.98, 95% CI =  0.42 to 2.30, P =  0.78, I² =  0%) (Fig 8).

## Discussion

Although some hypotheses about the mechanism of OAB have been proposed and accepted, [12,13] the exact pathogenesis of OAB remains unclear, and treatment for OAB still primarily focuses on symptom relief. Traditionally, anticholinergic agents were used for OAB treatment. [14] Mirabegron is the first β3-AR agonist approved for OAB treatment. [15] It works by selectively activating β3 receptors in the urothelium and bladder smooth muscle, increasing intracellular cyclic adenosine monophosphate (cAMP) levels. This, in turn, activates protein kinase A (PKA), which reduces calcium ion influx and inhibits detrusor muscle contraction, thereby relaxing the bladder smooth muscle and alleviating the clinical symptoms of OAB. [6] Vibegron is an effective selective β3-AR,with over 9000 times higher selectivity for β3-AR compared to β1-AR or β2-AR. [16] Some studies have indirectly compared the safety and efficacy of mirabegron and vibegron. They suggest that vibegron is more effective in reducing average urine volume [17].

We designed this head-to-head study to compare the efficacy and safety of the two medications, mirabegron and vibegron. Our results indicate that there are no significant statistical differences between the two drugs in relieving OAB symptoms. This is consistent with some earlier small-scale individual studies, [10] suggesting that both mirabegron and vibegron are effective and safe options.

We used OABSS to compare the efficacy results across the studies. Symptoms assessed, including frequency, urgency, nocturia, and incontinence, showed no significant differences, indicating similar efficacy between the two drugs. We believe that the two drugs have similar mechanisms of action, resulting in comparable effects on these symptoms. Additionally, the analysis of adverse effects, particularly common side effects such as constipation and dry mouth, also revealed no notable differences, further supporting the similarity in safety between the two medications.

Although mirabegron and vibegron show similar safety and efficacy in treating OAB, clinicians should select medications based on individual characteristics. This study aimed to statistically compare the efficacy of the two drugs using patient-reported outcomes and objective parameters such as OABSS and PVR, but no significant differences were found between the two treatments. clinicians should consider specific clinical situations, patient needs, drug costs, administration methods, sensitivity to the drug, and patient tolerance when making choices.

Additionally, since OAB patients require long-term medication for symptom control, future research should focus more on the long-term safety of these drugs. The current study’s follow-up period of 8 to 12 weeks does not fully assess potential risks associated with long-term use. Given that different patient populations may have varying tolerances to drug side effects, future studies should further explore adverse reactions in different subgroups.

This study is a meta-analysis that includes three studies, all of which used a 50mg dose of mirabegron and 50mg dose of vibegron. Although the typical clinical dose of vibegron is 75mg, the studies selected 50mg to evaluate its efficacy and safety at a lower dose. Given the consistency across these studies, they adopted the same dosages to ensure comparability of results. The 50mg dose of mirabegron has been shown to significantly reduce urinary incontinence and urgency episodes with a lower incidence of side effects, such as dry mouth, compared to traditional anticholinergic medications. [18] Therefore, this dosage selection is well-supported by existing evidence.

Although hypertension is a potential side effect of β3 agonists, it was only mentioned in one of the three studies included in our meta-analysis. In that study, only one patient experienced elevated blood pressure, while the other two studies did not report any blood pressure-related adverse events. As a result, we did not consider hypertension as a prominent or consistent side effect in our analysis. However, the potential for hypertension as an adverse effect should be noted, and further research is needed to investigate its frequency and clinical significance in larger patient populations.

The limitations of this study are primarily reflected in several aspects. First, the sample size of the included RCTs is relatively small (368 patients), and the follow-up period for all studies is short (8 to 12 weeks), making it impossible to thoroughly assess the long-term efficacy and safety of the medications. Additionally, the implementation of blinding in all studies was not strict; three RCTs did not adhere to rigorous double-blinding, and there was no information on whether the physician was blinded or whether patients were blinded. The study also did not adequately consider other potential influencing factors, such as patient comorbidities and adjustments in drug dosage, which might contribute to differences in drug efficacy and safety evaluation. Furthermore, all three included RCTs were conducted in Japan, limiting the generalizability of the findings. It must be acknowledged that this limitation may restrict the applicability of the results to diverse demographic and clinical settings.

In this study, due to the oversight of missing data during the literature screening and analysis process, we were unable to assess key clinical parameters such as the daily frequency of urination and urine volume. Although these physiological parameters are crucial in the evaluation of OAB, we did not include these data primarily because we did not fully recognize their absence during the screening phase. This oversight serves as a reminder that future systematic reviews should pay closer attention to the comprehensive collection of these core clinical parameters to ensure data consistency and completeness. Parameters such as urination frequency and volume are valuable for evaluating the efficacy of OAB treatments. However, due to differences in research design and measurement methods, collecting and integrating these data may present challenges. Therefore, we recommend that future studies place greater emphasis on systematically recording these physiological parameters to provide more comprehensive and accurate evidence for clinical decision-making.

The meta-analysis exhibited low heterogeneity (with I² values generally below 25%), indicating a high degree of homogeneity among the included studies and suggesting a high level of credibility for the results. However, low heterogeneity might also suggest insufficient diversity among the studies, particularly in the evaluation of efficacy and safety across different subgroups (such as males and females, and patients of different age groups). Since the majority of OAB patients are female, this study predominantly focused on female patients. Therefore, future research should include a broader population, especially evaluating the treatment effects in male patients, to further expand the scope of our findings.

We found no significant statistical differences between mirabegron and vibegron in terms of efficacy and safety; however, further high-quality RCTs are needed to confirm this conclusion. Future research should focus on the following aspects: (1) longer follow-up periods to assess the long-term safety and efficacy of the medications; (2) larger patient cohorts, particularly subgroup analyses of different age groups, genders, races, and patients with comorbidities; (3) exploring the efficacy and safety of the two drugs at different doses to determine the optimal dosing regimen; (4) cost-effectiveness analyses in different countries or regions to assist clinicians and policymakers in selecting the most appropriate treatment options.

## Conclusion

The use of mirabegron or vibegron does not appear to show statistically significant differences in efficacy and safety for OAB patients. Further high-quality prospective studies are needed to confirm this result. Clinicians should select the most appropriate medication based on the individual characteristics of the patient. Future research should focus on high-quality prospective studies, especially those addressing long-term use and different subgroups, to further clarify the optimal application of these two drugs in the treatment of OAB.

## Supporting information

Supplement 1A Funnel plot of the mean changes in OABSS before and after treatment. (TIF)

Supplement 1B Funnel plot of RR of adverse events after medication. (TIF)
