# Supplementary figures and images for: Comparison of efficacy and safety of mirabegron and vibegron in the treatment of Overactive Bladder (OAB) in older women: A systematic review and meta-analysis
Source: PLoS One. 2025 Apr 8;20(4):e0317550. doi: 10.1371/journal.pone.0317550 (PMC11977964; doi:10.1371/journal.pone.0317550)

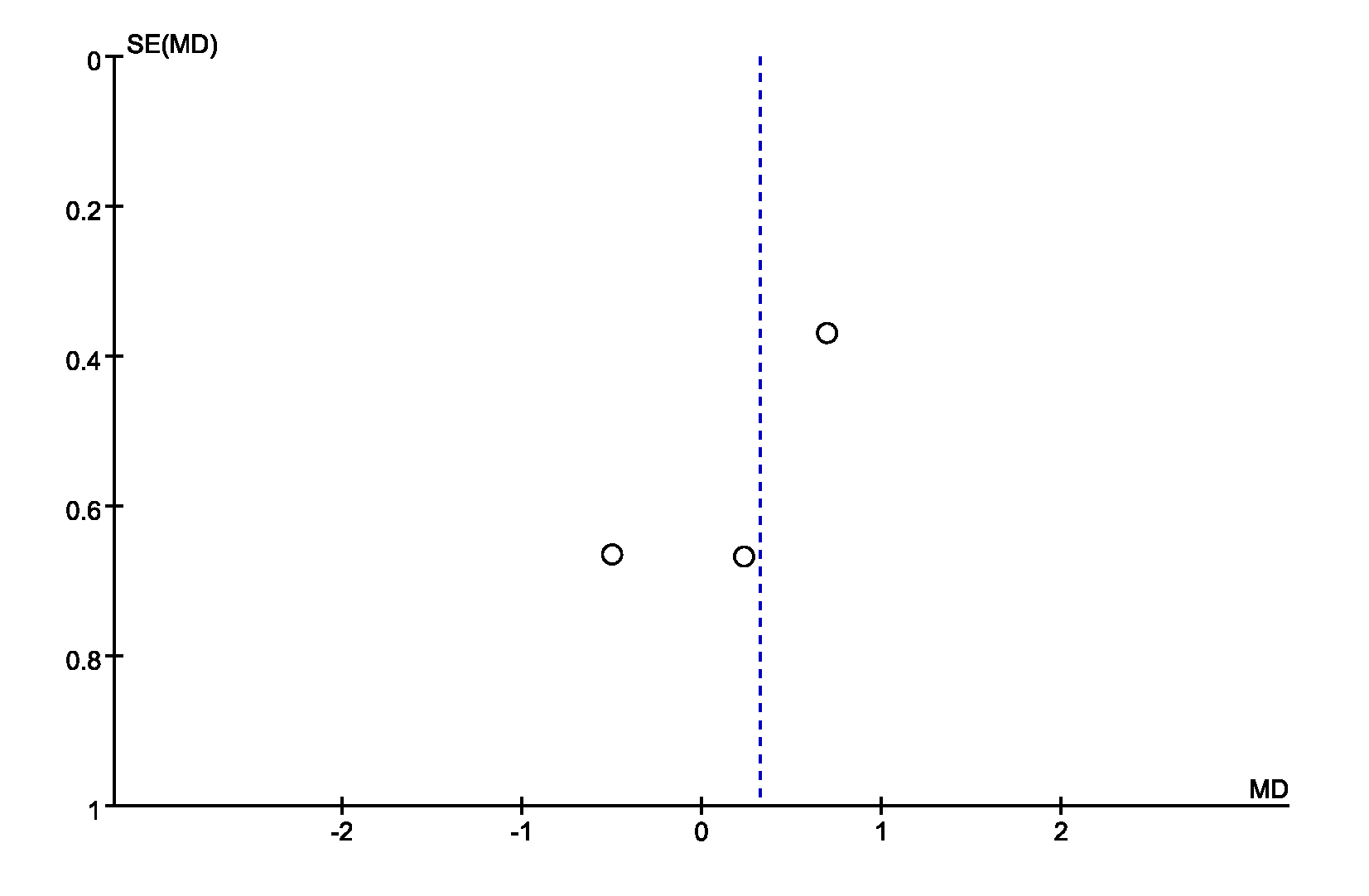

Supplement: Supplement 1A — (TIF) [file pone.0317550.s001.tif]

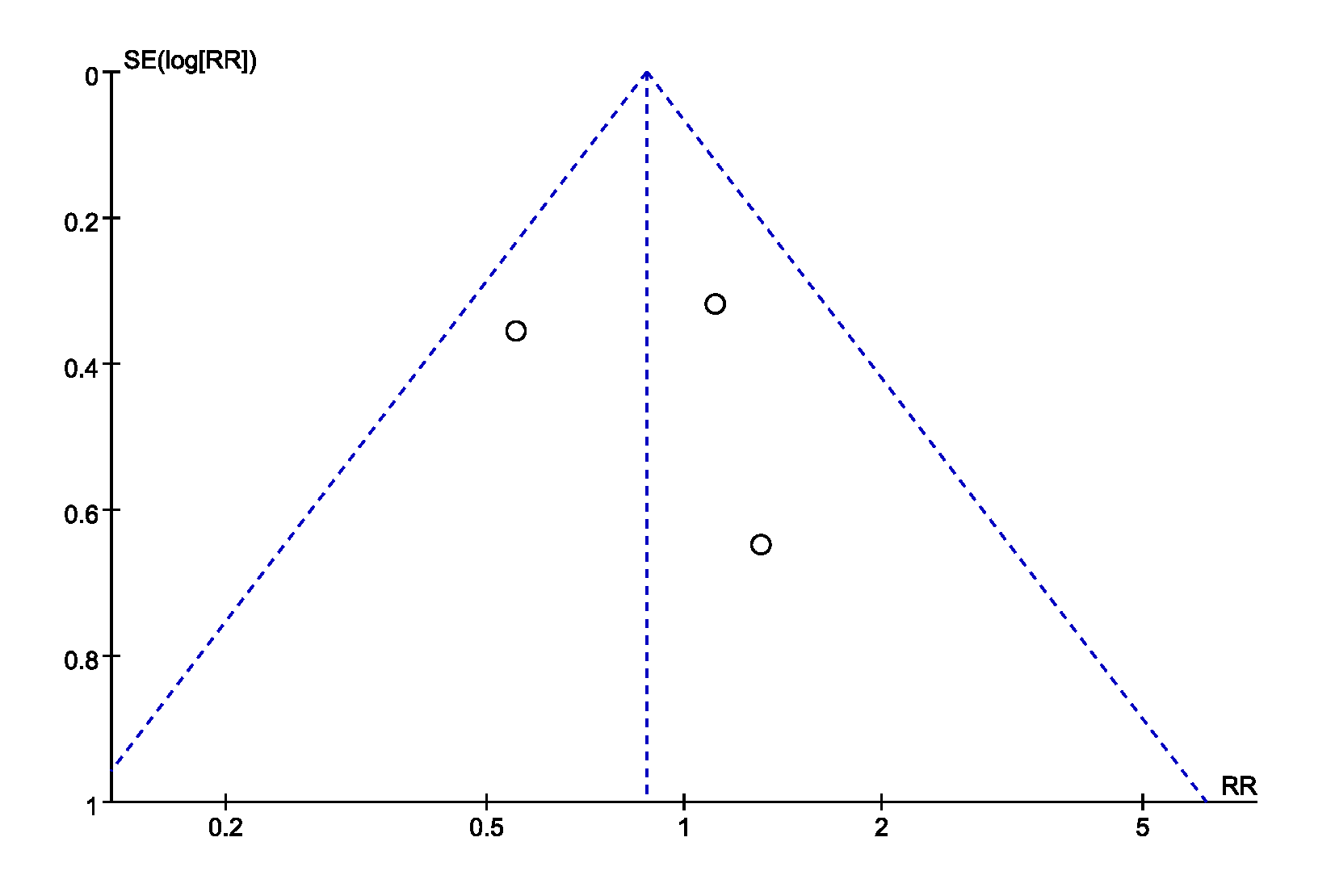

Supplement: Supplement 1B — (TIF) [file pone.0317550.s002.tif]
